# Role of Oxidative Stress in Metabolic Reprogramming of Brain Cancer

**DOI:** 10.3390/cancers15204920

**Published:** 2023-10-10

**Authors:** Kirti Agrawal, Shailendra Asthana, Dhruv Kumar

**Affiliations:** 1 School of Health Sciences and Technology (SoHST), UPES, Dehradun 248007, India; 2 Translational Health Science and Technology Institute (THSTI), Faridabad 121001, India

**Keywords:** brain cancer, oxidative stress, ROS, RNS, RONS, tumor microenvironment

## Abstract

**Simple Summary:**

According to Globocan 2020 data, approximately 308,102 new cases of brain cancer and central nervous system (CNS) cancers were reported worldwide, with 251,329 reported deaths. Reactive oxygen and nitrogen species (RONS) alter various signaling pathways to regulate cell stability. In this review, we will discuss the altered metabolism in brain cancer and emphasize the role of reactive oxygen species (ROS), reactive nitrogen species (RNS), and the tumor microenvironment in controlling metabolic shifts in brain cancer. We will also highlight potential therapies for targeting ROS, inhibiting cancer cell proliferation, and addressing gliomal stem-like cells.

**Abstract:**

Brain cancer is known as one of the deadliest cancers globally. One of the causative factors is the imbalance between oxidative and antioxidant activities in the body, which is referred to as oxidative stress (OS). As part of regular metabolism, oxygen is reduced by electrons, resulting in the creation of numerous reactive oxygen species (ROS). Inflammation is intricately associated with the generation of OS, leading to the increased production and accumulation of reactive oxygen and nitrogen species (RONS). Glioma stands out as one of the most common malignant tumors affecting the central nervous system (CNS), characterized by changes in the redox balance. Brain cancer cells exhibit inherent resistance to most conventional treatments, primarily due to the distinctive tumor microenvironment. Oxidative stress (OS) plays a crucial role in the development of various brain-related malignancies, such as glioblastoma multiforme (GBM) and medulloblastoma, where OS significantly disrupts the normal homeostasis of the brain. In this review, we provide in-depth descriptions of prospective targets and therapeutics, along with an assessment of OS and its impact on brain cancer metabolism. We also discuss targeted therapies.

## 1. Introduction

Brain tumors are the prevalent malignancies of the CNS. Many of these malignancies are difficult to treat and have a high fatality rate [1]. Brain tumors are categorized as gliomas (astrocytomas, ependymomas, oligodendrogliomas) and non-glial tumors (meningiomas, medulloblastomas). Based on the histology, these tumors are divided into four grades (grades I, II, III, and IV). Grade I cancers have a modest proliferating potency and can be treated with surgery. Grade II tumors have considerable permeation and relapse rates after the treatment. Grade III tumors have shown greater atypical mitosis.; Grade IV tumors show vascular proliferation and necrosis and are the most aggressive tumors [2,3]. Glioblastoma (GBM) is a type of grade IV brain tumor, which accounts for 75–80% of all brain malignancies. The CNS is a key location for metastatic tumors from different body parts to lodge [4]. Non-brain malignancies, including breast cancer and melanoma, ultimately spread to the brain in about 40% of cases [5].

Oxidative stress, characterized by an excess of ROS, has been linked to various health conditions, such as cardiovascular diseases, neurodegenerative disorders, diabetes, and a range of other illnesses [6]. These findings highlight the need for striking a balance among the comparative abundance of ROS and antioxidants. To maintain this equilibrium, cells have complicated biochemical and genetic pathways, and it is evident that their disruption can have serious pathological repercussions. Although ROS are protumorigenic, excessive levels of ROS are toxic at the cellular level, and tumor cells have shown an anomalous redox homeostasis [7]. Cancer cells have progressed to thrive in conditions wherever the oxidative load thrusts the redox equilibrium from the reduced state; tumor cells attain this state by increasing their antioxidant level to elevate ROS-driven cell proliferation to avoid the ROS threshold, which can activate apoptosis, ferroptosis, or senescence [8]. The brain is highly susceptible to OS in comparison to other organs [9]. It utilizes 15–20% of the total energy created by the body but accounts for only 2–3% of the body mass. The higher amount of PUFAs (omega-3 poly-unsaturated fatty acids) in the CNS is responsible for the increased mass-specific metabolism rate. Polyunsaturated fatty acids (PUFAs) constitute approximately 0.5–0.7% of the overall brain mass. They are especially vulnerable to an uncontrolled form of oxidative stress, wherein hydroxyl radicals (•OH) remove a bis-allylic hydrogen, subsequently introducing molecular oxygen to generate a lipid peroxyl radical. These peroxyl radicals can then be transformed into peroxides and undergo spontaneous decomposition. If the oxidative breakdown of PUFAs in the brain is primarily initiated by the direct action of •OH radicals, it implies that a single •OH radical should lead to the oxidative degradation of one PUFA molecule. Given that the majority of brain PUFAs are either arachidonate (ARA, ω3) or docosahexaenoate (DHA, ω6), it can be deduced that a reduction in the concentration of one of these PUFA species should result in a decrease in reactions between this specific PUFA and the available •OH radicals. Consequently, more •OH radicals become accessible to enhance the oxidative degradation of the other PUFA species [10]. Additionally, the brain has significant quantities of redox-active iron and copper, making it more vulnerable to OS. One of the primary mechanisms underlying the carcinogenic properties of metals involves the capacity of transition metal ions to trigger oxidative stress. Ferroptosis is a cellular death pathway believed to be triggered by a combination of two mechanisms influencing the cell—the impairment of the cell’s antioxidant defenses and the elevation of the intracellular iron levels. Additionally, iron is implicated in the formation of metastases and the growth of new blood vessels (angiogenesis) through the activity of iron metalloproteases. Iron can influence the microenvironment by facilitating the degradation of the extracellular matrix, thereby impacting cancer metastasis. Furthermore, iron can stimulate the multiplication of cancerous cells. This is because cancer cells have heightened iron demands owing to their increased rates of proliferation and DNA synthesis [11]. Copper (Cu) accumulation is associated with enhanced cell growth and metastatic potential and the formation of new blood vessels. Prolonged exposure to elevated levels of copper in drinking water has been found to significantly boost the proliferation of cancer cells [12]. For instance, the formation of intracellular free ion Cu (I) can hinder enzyme activity by oxidizing cysteine residues in iron–sulfur cluster proteins or by reacting with hydrogen peroxide to form highly reactive hydroxyl radicals [13]. Hydroxyl radicals are potent oxidizers of DNA, leading to mutagenic changes and cell damage. Simultaneously, copper binds indiscriminately and directly to the thiol and amino groups of proteins, disrupting their structure and biological function. One such copper-dependent enzyme, LOX (amine oxidase), plays a role in promoting the invasion and migration of tumor cells [14]. In living cells, the generation of ROS is often attributed to Fenton and Haber–Weiss reactions. These reactions involve the decomposition of hydrogen peroxide (H_2_O_2_) with the involvement of metal ions such as iron (Fe) and copper (Cu). This process results in the creation of hydroxyl radicals (•OH) and hydroxyl anions (OH^−^) [15].

Because the brain primarily consists of critically differentiated neurons and glia cells, it has limited ability to repair injured cells [16]. High quantities of ROS, which are the moderately condensed metabolites of oxygen with significant oxidizing capacities, are toxic to cells, yet at lower concentrations they help complex signaling activities [17]. Homeostasis and cellular signaling require reactive oxygen species, which are generated as regular metabolic byproducts of cell. These are produced by certain oxidases of cell membranes in reaction to various growth factors (GFs) and cytokines. They also function as subordinate messengers for particular signaling pathways and have shown significant activation in gene regulation [18]. The cells engage in active defense to keep the levels of ROS normal, which includes changing antioxidant enzymes that alter free radicals into less harmful and stable compounds. Most of the cancer cells shows high metabolic activity and are hypoxic in nature [19]. As a result of their quick growth and limited vascularization, they generate more reactive oxygen species, which damage the DNA by dispersing through the membranes of mitochondria and act as signaling messengers in a variety of redox-sensitive signaling pathways, participating in cell survivability, proliferation, and therapeutic resistance [20]. Because OS play a major role in hallmarks of cancer (including stemness, angiogenesis, metastasis, and invasiveness), decreasing OS using potent antioxidants has become a popular cancer prevention technique [21]. Furthermore, cancer cells acquire methods to counteract the increased oxidative stress. As a result, several cancer therapy techniques work by exposing tumor cells to apoptosis.

## 2. Function of ROS and RNS in the Brain

### 2.1. Reactive Oxygen Species (ROS)

ROS are vastly reactive oxygen molecules that contain a (OH) radical group, which has high reactivity, as well as species that have low reactivity such as superoxide (O_2_) and hydrogen peroxide (H_2_O_2_) [22,23]. ROS are produced in cells mostly due to the conversion of a lesser amount of oxygen delivered by the electrons that are seeped from the electron transfer chain (ETC) of mitochondria in the course of the oxidative phosphorylation process [24]. Incomplete molecular oxygen reduction via enzymatic interactions is a further source of ROS involving intra-cellular oxidases such as NADPH oxidase, xanthine oxidase, cyclo-oxygenases, lipo-oxygenases, and cytochrome-P450 reductases [25,26]. In the CNS, however, NADPH oxidase and ETC are the principal ROS producers. The enzymatic or non-enzymatic breakdown of ROS by catalase, glutathione peroxidase, thio-redoxin, superoxide dismutase (SOD), gluta-redoxin, peroxiredoxin, vitamins, urate, glutathione, ubiquinone, or lipoate balances their formation [22,27]. As a result, the homeostasis of cellular reactive oxygen species is based on the equilibrium among free radical degradation and production.

ROS are required for appropriate brain or cell activity in small levels. Furthermore, ROS upregulate many endogenous cytoprotective molecules, resulting in several alterations to prevent or neutralize oxidative stresses. Physiological levels of ROS are particularly important throughout brain development. ROS play a part in healthy neural–stem cell multiplication and differentiation in the developing brain, and neurons create great quantities of physiologically active ROS to support vital processes [28]. In a previous study, when neuron cell cultures were treated with antioxidants, the neural–stem cell (NSC) multiplication rate was significantly reduced and the differentiation of the neurons was altered. Hypoxia-conditioned medium, on the other hand, promotes NSC proliferation and favors neuron survivability over astrocyte survival [29]. In induced pluripotent stem cell (iPSC) neurospheres, standard culture air conditions affect proliferation, cell survival, and fate determination [30]. ROS control redox-sensitive proteins in the brain in adults, which permits the brain to execute physiological activities. Long-term potentiation (LTP) is stimulated by ROS secondary messenger-like carbon monoxide (CO) and nitrous oxide (NO), which induce GMP-regulated glutamate discharge [31]. High doses inhibit LTP and synaptic signaling, while low LTP concentrations promote these mechanisms. In cognitively healthy people, the OS levels, as evaluated by altered membrane lipids, oxidized proteins, and DNA damage, increase with age, whereas the natural antioxidant levels become dysregulated. The intrinsic defenses of the brain steadily begin to be overwhelmed by physiological levels of RNS or ROS [16]. The particular pathways that contribute to OS in the brain are complex, and oxidative damage accretion is not constant among the persons, brain regions, or even in various types of cells inside the brain.

Antioxidants have demonstrated their ability to interact with and neutralize free radicals, mitigating their detrimental effects. These effects encompass gene mutations, oxidative harm to chromosomes and proteins, lipid peroxidation of cellular membranes, and irregular cell growth [32]. The brain, constituting 20% of the body’s total metabolic activity and having a higher oxygen consumption rate compared to other tissues, is considered susceptible to oxidative stress damage, potentially fostering the development of brain cancer [33]. OS-induced damage to cellular macromolecules can lead to genomic instability, a factor that promotes cancer formation. ROS-mediated cell signaling events are implicated in various aspects of cancer cell biology, including cell proliferation, growth, differentiation, angiogenesis, and survival. The brain’s antioxidant capacity is notably lower compared to other tissues [34]. Within the brain, endogenous antioxidants encompass diverse groups of molecules, including both water-soluble and lipid-soluble enzymes. Examples of these enzymes include catalase (CAT), glutathione S-transferase (GST), glutathione peroxidase (GPx), glutathione reductase (GR), and superoxide dismutase (SOD). Additionally, certain cellular metabolites such as reduced glutathione (GSH) and thioredoxin contribute to the antioxidant defense system [35]. Numerous studies have highlighted the pivotal role of antioxidant systems such as SOD and thioredoxin in safeguarding cells from damage induced by ROS [36]. The potential chemo-preventive benefits of antioxidants have been documented in various studies [37,38]. However, the exact role of antioxidants in chemoprevention remains a subject of ongoing research and is not fully understood. It is worth noting that exogenous antioxidants can also promote pro-oxidant reactions, which are linked to the destruction of cancer cells through the induction of an excessive amount of ROS. Consequently, antioxidants may not only have a role in preventing the recurrence of cancer but could also complement cancer chemotherapy. The effectiveness of antioxidants, whether as pro-oxidants or antioxidants, depends on the dosage administered in therapy.

### 2.2. Reactive Nitrogen Species (RNS)

Nitric oxide (NO) is largely non-reactive, and NO and its byproduct peroxynitrite (ONOO), which can damage several biological molecules, are examples of RNS. A cluster of enzymes such as NOS (nitric oxide synthase) produces NO from its precursor, L-arginine. Inducible NOS (iNOS or NOS-II), neuronal NOS (nNOS or NOS-I), endothelial NOS (eNOS or NOS-III), and mitochondrial NOS (mtNOS) are the four participants of the NOS clan, which are found in the brain. NOS-I is extensively produced in neurons, while NOS-III is found in endothelial cells inside the brain vessels [39,40]. NOS-II is considered to be triggered in astrocytes, macrophages, or microglia by the stimulation of inflammation [41]. More evidence suggests that NOS-III is expressed in astrocytes, and NOS-II was found to be increased in neurons and microglia in a constitutive manner [42]. Further, mtNOS was discovered in the inner mitochondrial membrane, represented as the NOS-I isoform. The NO can be created locally in the mitochondria by mtNOS, and too much NO inhibits the action of complexes I and IV inside the ETC of mitochondria, resulting in the modulation of mitochondrial ROS [43,44,45]. The O_2_•^−^ reacts with NO 3–6 times quicker than SOD to generate the powerful oxidant peroxynitrite than it does with SOD to produce H_2_O_2_ [46]. Peroxynitrite can react with lipids, DNA bases (DNA strand breaks), thiols (thiol oxidation), amino acids (AAs), and low-molecular weight antioxidants, in addition to depleting NO bioavailability [22].

**Figure 1:**
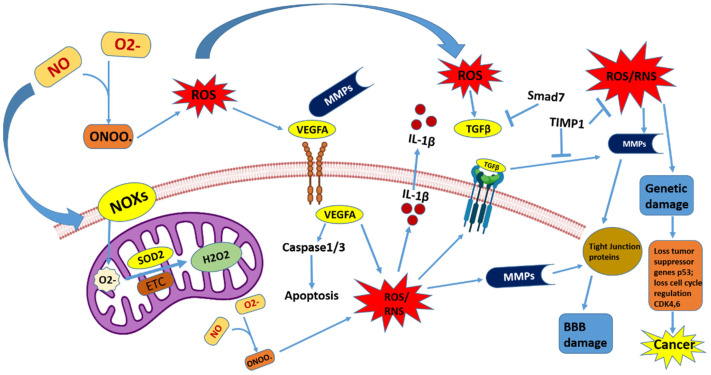
Role of ROS and RNS in genetic damage and BBB damage, causing oxidative stress and cancer in the brain. NOXs in reaction with O_2_^−^ produce ROS and RNS, in turn activating MMPs (matrix metalloproteinases), which damage the blood–brain barrier (BBB) and cause oxidative stress in the brain. ROS activate VEGFA and TGFβ, which leads in the formation of ROS and RNS. The antioxidant SOD2 catalyzes H_2_O_2_, which activates ROS AND RNS and also leads to genetic damage and a loss of the tumor suppressor (p53) and cell cycle regulators (CDK4,6), which leads to cancer activation. Smad7 and TIMP1 act as the inhibitors of ROS AND RNS activity. Abbreviation: ROS—reactive oxygen species; RNS—reactive nitrogen species; BBB—blood–brain barrier; ETC—electron transport chain; MMP—matrix metalloproteinase; SOD2—superoxide dismutase-2; VEGFA—vascular endothelial growth factor A; TGFβ—transforming growth factor-beta; Smad7—suppressor of mothers against decapentaplegic-7; TIMP1—tissue inhibitor of metalloproteinase.

The reactive oxygen and nitrogen species (RONS), which are extremely reactive radicals with the unpaired valence shell electrons, show their chief role in the innate immune system. Macrophages emit superoxide O_2_ and H_2_O_2_ when stimulated by cytokines via a NADPH-dependent oxidative surge [47]. Macrophages also produce NO, a signaling chemical involved in vascular tone modulation that can interact with oxygen to form peroxynitrite (ONOO^−^) [48]. The H_2_O_2_ can be combined with halogens by neutrophils and eosinophils to produce hypochlorous acid (HOCl) and hypobromous acid (HOBr) [49]. These reactive oxygen, halogen, and nitrogen species in reaction with amino acids, nucleic acids, and lipids produce a diverse range of reactive products, which can lead to genomic instability. The role of ROS and RNS is illustrated in Figure 1.

## 3. Mitochondrial ROS in the Metabolism of Brain Cancer

Mitochondria are the primary generators of intracellular ROS, as a small percentage of the oxygen that is used for OXPHOS can be transformed into anion superoxide. The additional enzymes of mitochondria implicated in ROS formation include pyruvate de-hydrogenase (PDH), a-ketoglutarate-de-hydrogenase (a-KGDH), acylCoA de-hydrogenase, and glycerol-3-phosphate de-hydrogenase [50]. The mitochondrial ROS (mROS) levels are tightly regulated in normal cells and show a role in several cell mechanisms such as autophagy, differentiation, and metabolic adaptation. Cancer cell activation, losses of tumor suppressors, tumor-induced mutations in the enzymes of the TCA cycle, and hypoxia cause aberrant levels of mROS, which operate as retrograde signals to keep cancer cells alive [51]. By initiating the mutations in nuclear DNA or mitochondrial DNA (mtDNA) through disturbing many biological mechanisms such as cell multiplication, apoptotic resistance, and metabolic re-programming, mROS stimulate all stages of cancer from tumor activation to cancer cell proliferation and metastasis [52]. Moreover, mROS can initiate a variety of potential carcinogenic signaling pathways, including the EGFR signaling system and the Akt/NF-kB-dependent mitochondrial transcription factor B2 (TFBM2) signaling pathway, both of which have been linked to cancer proliferation [53]. Several signal transduction pathways involved with metastatic spreading, such as protein tyrosine kinase-2 beta (PTK2B) and Src signaling, can be triggered by mROS [54]. In melanoma and lung cancer, oxidative stress, on the other hand, has been shown to restrict metastatic spread in multiple investigations. To escape ROS-driven mitochondrial permeability transition (MPT)-regulated apoptosis, cancer cells regulate large quantities of antioxidant proteins to inhibit ROS buildup and may steer tumor cells nearly to a proliferation state. The cancer cells can stay in the tumor range of ROS levels if there is a decent equilibrium between mROS creation and ROS hunting. In this regard, nuclear factor (erythroid-derived 2)-like 2 (Nrf2) is a transcriptional factor that binds to the DNA and is well-known to be the principal controller of genes that alleviate OS [55]. The mROS causes the allosteric inhibitor, kelch-like ECH-associated protein I, to be degraded, causing the immediate activation of Nrf2 (KEAP1). Although Nrf2 was once thought to be a tumor suppressor, new research has revealed that it has a protumoral function that not merely imparts OS resistance but furthermore regulates ROS generation via NADPH oxidase & stimulates cancer-related metabolic pathways. As a result, in various forms of cancer, the constitutive stabilization and activation of Nrf2 has been linked to a bad prognosis [56]. Hypoxia-inducible factor-1 (HIF-1) stimulation follows mROS formation, which derives a metabolic alteration from OXPHOS to glycolysis by upregulating glycolytic enzyme expression, facilitating cancer formation and metastasis [57]. The relationship between HIF-1 and mROS seems to be complicated, as excessive mROS activates HIF-1, although stimulation of the glycolytic program alleviates OS as compensation. HIF-1 reduces mROS generation, enhances tumor progression, and aids metastatic cell survivability in numerous kinds of cancer, which supports this theory [58]. Overall, our findings imply that the practical role of mROS varies based on the category and stage of tumor, although mROS selectively activates distinct signaling pathways that lead to protumoral metabolic reprogramming. As a result, anticancer therapies that targets mROS and antioxidant mechanisms could be advantageous.

## 4. Metabolic Reprogramming in Brain Cancer and Microenvironmental Stress

Metabolic reprogramming may have an impact not only on cancer cells’ survival and proliferative signals but also on the cancer microenvironment. The tumor microenvironment is complex, encompassing both social and physical surroundings. Fibroblast cells, endothelial cells, immune cells, the extracellular matrix (ECM), and bacteria make up the social microenvironment. All entities existing in the social microenvironment will face food deprivation and oxygen unavailability, low pH levels, and redox stress during tumor initiation and progression, which together make up the physical microenvironment [59]. The pyruvate dehydrogenase complex in mitochondria transforms glucose-derived pyruvate to acetyl-CoA, although hypoxia slows this procedure by increasing LDHA and PDK1 [60,61]. Lactate buildup is caused by continuously engaged glycolytic and LDHA enzymes, resulting in a low-pH microenvironment throughout tumor growth. Hypoxia also influences lipid metabolism by inhibiting fatty acid oxidation, which is reduced by HIF1. Nutrient deprivation is another common microenvironmental stressor for cancer cells [62]. Tumor cells undergo metabolic reprogramming in response to nutritional deprivation, allowing them to use all available energy to sustain their own growth.

### 4.1. Aerobic Glycolysis and Lipogenesis in Medulloblastoma

Although aerobic glycolysis and lipogenesis have been examined the most in Shh-driven medulloblastoma, these metabolic processes may also be shared by all medulloblastoma sub-groups. Outside of the brain, stimulation of the WNT signaling pathway promotes glycolysis as an alteration with the OXPHOS in normal and altered cells. Triggering the MYC can be crucial and defines the metabolism in medulloblastomas across their sub-groups, according to numerous lines of evidence. MycN drives aerobic glycolysis in CGNPs and Shh-driven medulloblastomas in Smo M2 mice by acting downstream of Shh [63,64]. The WNT signaling may enhance glycolysis partly through the activation of MYC, although whether the link exists in WNT subgroup medulloblastomas is unknown. MYC family amplifications are linked to group 3 medulloblastomas, and MycN overexpression in mice results in medulloblastomas that have a gene expression pattern similar to group 3 tumors [65,66]. While glucose metabolism in group 3 medulloblastomas and MycN-driven medulloblastomas in mice have yet to be investigated, it is likely that MYC reconfigures the metabolism to favor glycolysis in these tumors, as it does in other malignancies. Glioblastomas, like medulloblastomas, express HK2 and show significant glucose uptake in FDG PET investigations, implying that aerobic glycolysis is important in malignant brain tumors with a variety of pathologies [67]. Various malignant brain tumors mimic the metabolic adaptations of neural progenitors by activating lipogenesis and aerobic glycolysis.

### 4.2. Metabolism in Glioblastomas (GBMs)

**Figure 2:**
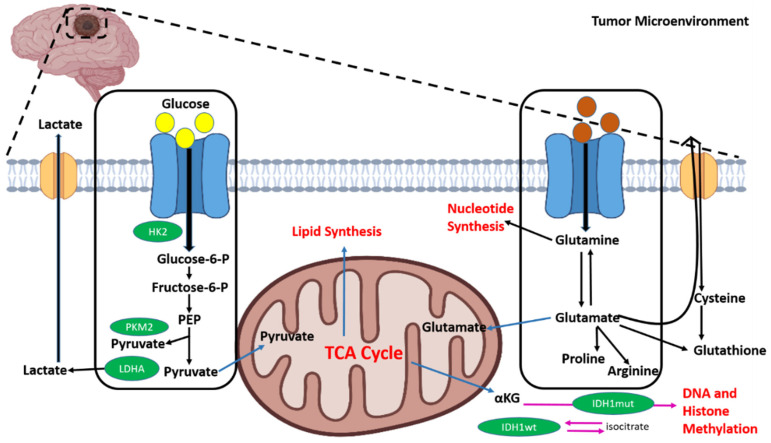
Metabolic programming in glioblastoma cells. Glycolysis and glutaminolysis accelerate the TCA cycle in mitochondria and the IDH catalyze the oxidative decarboxylation of isocitrate to create CO_2_ and αKG.

Since 2016, the WHO has divided GBMs into two types: (i) IDH-mutant glioblastomas (mGBMs) and (ii) IDH-wildtype glioblastomas (wGBMs). In the TCA cycle, the IDH catalyze the oxidative decarboxylation of iso-citrate to create carbon dioxide and alpha-ketoglutarate (α-KG). A wGBM is usually a novel tumor, whereas the mGBM is caused by a lower grade glioma (LGG), implying that the GBM has a metabolic problem [68]. Additionally, in the existence of oxygen, tumor cell “respiration” is impeded according to Warburg. To increase tumorigenic characteristics via glycolysis, cancer cells upregulate GLUT3 and selectively absorb nutrients from the extracellular environment. This phenomenon is called “aerobic glycolysis.” Cancer cells, on the other hand, can survive in a glucose-depleted environment by experiencing a metabolic rewiring, which permits their proliferation and infiltration and allows them to defy cancer therapies [69]. Certainly, the GBM cells seem to be extremely malleable in response to alterations in nutritional sources, and the metabolism in GBM tumors is illustrated in Figure 2. A multiomic study aided in the creation of a new categorization system for GBMs, dividing them into four subgroups based on developmental and metabolic characteristics. The mGBM sub-type primarily comprises oxidative tumor cells that rely on oxidative phosphorylation rather than glycolysis (OXPHOS) [70,71]. The “reverse Warburg effect” is the term for this. The Warburg and reverse Warburg effect are the adaptive processes that cells use in response to the cell cycle and tumor microenvironment composition [72]. During early embryogenesis, when the oxygen supply is minimal, the Warburg effect happens in normal cells. Additionally, tumor cells with OXPHOS often show a less used glycolysis pathway and ATP synthesis. In this situation, glutaminolysis would aid in the formation of high-energy phosphate [73]. When OXPHOS is turned off, glutamine-derived succinate offers enough ATP via mitochondrial substrate-level phosphorylation (mSLP) to keep the glioblastoma tumor growing [74].

### 4.3. Inflammation-Regulated Microenvironment in Brain Cancer

The brain tumor microenvironment is made up of a variety of cells, comprising infiltrate inflammatory cells; stem-like cells; and cells with neuronal, myeloid, and glial characteristics. Excessive tumor growth induces numerous infiltrative immune cells such as eosinophils, T-lymphocytes, macrophages, and neutrophils, resulting in hypoxia and aberrant vascular proliferation. In the gliomal niche, two kinds of astrocytes (A1 and A2) that contribute to inflammatory response have been discovered [75]. While the A2 sub-type promotes neuronal survivability and connection under the state of oxygen deficiency, the A1 astrocytes are neurotoxic in nature and are mostly generated in reaction to damaged and pro-inflammatory stimuli. It has been claimed that an interaction between reactive A1 astrocytes and inhabitant gliomal cells promotes localized inflammatory effects. There is a multifaceted interplay between these many cell types, which produce a variety of cytokines [76]. These cytokines act in an autocrine and paracrine fashion, activating or suppressing a variety of signaling pathways that were originally designed to aid tissue restoration. However, the tumor microenvironment’s chronic inflammatory status influences the signals in habitats that stimulate tumor proliferation and spread. The inflammatory mediators’ primary job is to flush out any undesirable cell and stimulate fibrous tissue development to repair the wound area. Inflammatory cells, on the other hand, respond inversely to prolonged damage, releasing a large number of cytokines and other immunosuppressants [49]. Likewise, immune cells that take part in tumor infiltration, such as natural killer (NK) cells and cytotoxic T-cells, begin to produce inflammation-responsive mediators rather than demonstrating tumor suppression. This results in precancerous lesions, and the inflammatory milieu causes de-regulated immune responses and maintains tumor growth. The two common components of the brain tumor microenvironment are microglial cells and tumor-associated macrophages (TAM), which gather inside and nearby to the gliomal mass and collaborate in tumor cell invasion and proliferation. Various cytokines and chemoattractant factors, such as hepatocyte growth factor (HGF) and scatter factor (SF), monocyte chemo-attractant proteins 1 and 3 (MCP-1, MCP-3), macrophage colony-stimulating factor 1 (CSF-1), granulocyte macrophage colony-stimulating factor (GM-CSF), and glial-cell-derived neurotrophic factor (GCDF) [77]. Several cytokines and GFs (interleukin (IL)-6, IL-1, epidermal growth factor (EGF), transforming growth factor (TGF), and stress-inducible protein-1 (STI1)) are released by glioma-associated microglia and macrophages, which aid in tumor proliferation and migration. TGF-2 stimulates the extracellular-matrix-degrading enzymes matrix metalloprotease (MMP)-2 and MMP-9, which are activated by membrane type 1 matrix metalloproteinase (MT1-MMP) and urokinase plasminogen activator (uPA) to increase gliomal invasion. MMPs are released as inactive zymogens by gliomal cells, and the enhanced production of these enzymes has been frequently reported in this tumor type [77,78]. Myeloid-derived suppressor cells (MDSCs) mediate immunosuppression in the gliomal microenvironment by interacting with the activation of cytotoxic T-cells. Moreover, MDSCs can enhance angiogenesis and metastasis by secreting a variety of substances such as GM-CSF and VEGF. Other cell types (adaptive immune cells, leukocytes, and humoral cells) that interact with glioma cells have been discovered in the brain tumor niche [79]. Immune-inhibitory cytokines help to keep the tumor location immune-suppressive. Furthermore, persistent tumor growth can cause hypoxia and abnormal vascular proliferation, which can cause the production of various inflammatory mediators and enhanced microglial stimulation. The inflammation-responsive mediators such as T-cells and tumor-related macrophages aid in the spread of breast cancer cells, NSCLCs, and melanoma cells into the brain. Brain metastasis initiation cells must first dislocate from their usual sites and further invade the systemic circulation before they may spread [80]. The occurrence of macrophages in the tumor microenvironment promotes tumor cell intravasation by forming actin-rich deprivation protrusions, which further leads to matrix barrier degradation. Cancer cells can safely travel through the circulation thanks to an unregulated immune response caused by inflammation. By encouraging linkages to the brain capillaries and successive relocation by the microvascular endothelial cells, local chemokines and cytokines released by the pre-existing inflammatory brain contribute to the cancer cells’ brain-specific invasive activity. Inflammatory macrophages and MDSCs aid in extravasation by infiltrating tumor cells, encouraging angiogenesis and ECM remodeling [81,82].

### 4.4. Role of Microenvionment in GBM

Cancer-induced metabolic changes in the microenvironment are either important for tumor maintenance or may play a role in carcinogenesis. Surprisingly, OS appears to be at the heart of metabolic rewiring. ROS are released by cancer cells and diffuse to stromal cells, causing oxidative stress [83]. Oxidative stress triggers HIF-1 and nuclear factor kappa-light chain enhancer, causing a metabolic change in activated B-cells (NF-kBs). These transcription factors (TcFs) promote angiogenesis and perivascular niche conversion to boost oxygen availability. The secretion of higher energy substrates such as lactate and pyruvate causes aerobic glycolysis, mitochondrial dysfunction, lysosomal breakdown, and autophagy. These chemicals would be taken up by proliferative cancer stem cells and used for OXPHOS but the reverse Warburg effect takes place [83]. GBM-associated stromal cells (GASCs) act similarly to CAFs, which have been found in the stroma of carcinomas and have been shown to promote tumor growth in vivo and in vitro [84]. In contact with GBM, stromal cells such as non-neoplastic astrocytes could transport mtDNA and mitochondria through the connexin (Cx) family proteins and gap junction channels, as well as sub-cellular transfer processes such as the tunneling of nanotubes and microvesicles, presenting their chief role in GBM progression, which is amplified by ECM enriched in hyaluronic acid [85]. The physiological neuron–glia interaction is comparable to the metabolic coupling. Neurons are basically oxidative cells with lactate absorption, whereas astrocytes and microglia have higher glycolytic rates and secrete lactate, allowing neurons to meet their needs. Regulating the PDH activity, on the other hand, can reverse the metabolic differentiation patterns [86].

## 5. Targeting ROS and Glioma Stem-like Cells for Cancer Cell Death

### 5.1. Upregulation of ROS

The antitumorigenic signaling of ROS can be used for tumor treatment by increasing the ROS to toxic levels and draining the antioxidant system’s ability, resulting in programmed cell apoptosis. Anthracyclines, cisplatin, bleomycin, and arsenic trioxide, among other chemotherapy medicines, stimulate ROS generation, causing irreversible damage and cell apoptosis, a method used to treat acute lymphoblastic leukemia (ALL) [87]. Daunorubicin is an anthracycline that causes enhanced sphingomyelinase and ceramide activation, which leads to JNK pathway activation and apoptosis. In the presence of decreased NADPH, it combines with cytochrome-p450 reductase to create intermediates of semiquinone radicals, which further decreases O_2_ to form O_2_^−^ [88]. Doxorubicin is a broadly used anthracycline in the treatment of cancers such as breast, liver, bile duct, esophageal carcinoma, endometrial carcinoma, pancreatic, Kaposi’s sarcoma, gastric, osteosarcoma, Hodgins and non-Hodgins lymphoma, and soft tissue sarcoma cancers [89]. This increases the ROS production, which in turn activates the sulindac, a non-steroidal anti-inflammatory medication (NSAID) that operates by increasing ROS generation, which has been used to treat colon and lung cancers. It causes harm to the mitochondrial membrane, making tumor cells more vulnerable to H_2_O_2_—induced apoptosis [90]. Tumor cells adapt to OS by increasing the glucose metabolism, which prevents cell death by redox-inactivating cytochrome c.

### 5.2. Targeting Cancer Cell Proliferation by Downregulating ROS

Reduced reactive oxygen species level mean much fewer metabolic alterations, downregulated levels of DNA damage, and genetic instability, thereby causing reduced cell proliferation and survival. Therefore, ROS generation in tumor cells can be controlled to diminish protumorigenic signaling. Metformin is a drug used to treat type-2 diabetes and inhibit complex I of the mitochondrial ETC, which is linked to reductions in cancer occurrence and death. Metformin promotes apoptosis by boosting MnSOD/SOD2 protein expression while decreasing NOX2/NOX4 protein expression [91]. NOX4-produced ROS can be inhibited by the flavo-protein inhibitor diphenylene iodonium (DPI). As a result, this induces apoptotic cell death via the AKT–apoptosis signal regulating kinase-1 (ASK1) pathway, reducing protumorigenic effects in many malignancies [92]. Furthermore, suppression of tyrosine kinase FLT3-ITD, p22phox, and NOX4 activity in acute myeloid leukemia (AML) cells leads to reduced cell survivability and reductions in DNA damage and genomic instability according to the research. Antioxidants are also supposed to inhibit ROS generation, although this is debatable [93].

### 5.3. Targeting Stem-like Gliomal Cells

Through epigenetic regulation, the Wnt signaling pathway is abnormally active in glioblastoma multiforme. Because of the influence of Wnt signaling on stem cell characteristics, this change in signaling is thought to accelerate glioblastoma invasion and proliferation [94]. In gliomal cells, COX inhibition reduces cell proliferation, motility, and colony formation, and some of these effects may be due to altered Wnt–β-catenin signaling [95]. The metabolomic targeting of GSC is increasingly being studied as a cancer therapy. Inhibiting fatty acid synthase (FASN) enzyme has been shown to decrease lipogenesis, and as a result the expression of glioblastoma stem cell (GSC) markers such as CD133, SOX2, and nestin, as well as cell proliferation and migration. In contrast to glycolytic bulk tumor cells, the GSC metabolism is expected to rely mainly on OXPHOS [96]. Dichloroacetate, which inhibits pyruvate de-hydrogenase kinase-1, causes a GSC metabolism shift from OXPHOS to aerobic glycolysis, resulting in apoptotic stimulation [96]. In recent years, the use of diet interference to treat glioma has gained traction, and multiple clinical trials are investigating the effectiveness of the keto diet in combination with conventional treatments such as TMZ for the treatment of GBM. The keto diet, which involves higher fat levels and low levels of carbohydrates, relies on the capability of normal cells to adjust to decreasing glucose availability by boosting their capacity to utilize ketone bodies as an energy source [97]. Tumor cells, on the other hand, are ill-equipped to adjust to the metabolic stress, which could make them more susceptible to chemotherapeutic therapies. GSC stemness is thought to be affected by IDH1 and IDH2 mutations in the glioma. This is because the oncometabolite R-2-HG has a major effect on the epigenome, causing hypermethylation of the genes involved in differentiation. Under hypoxic conditions, the S-enantiomer of 2-HG (S 2-HG) generated by glioma cells with normal wild-type IDH is thought to have similar effects [98]. For patient therapy with IDH1 mutant tumors, the advancement of new IDH1 inhibitors, in addition to those now being explored, will be critical. The few FDA-approved drugs available for the therapy of high-grade gliomas are listed in Table 1.

## 6. Conclusions and Future Perspective

The numerous complicated linked factors that make the brain vulnerable to oxidative stress are unsaturated lipid accumulation, excessive glycolysis, mitochondrial glycolysis, calcium and glutamate production, limited antioxidant defense, redox active transition metals, RNA oxidation, and the auto-oxidation of neurotransmitters. Because it uses chemically different reactive species to carry out heterogeneous signaling processes, the brain is vulnerable to OS. By employing nitric oxide to adjust the synaptic elasticity, lipid radicals for ferroptosis signaling when lipid signaling stops working, or mitochondria [O_2_^−^/H_2_O_2_] to regulate hypoxia, the link among the species-specific beneficial and detrimental biochemistries is delicate, which makes the context of the brain bittersweet, where excellent redox signaling functionality readily results in OS when electrons go off track. With the help of targeting ROS or targeting glioma–stem cells, there is great potential for future advances, which supports the recent idea of integrative treatments focused intensively on the pathophysiology of OS in brain cancer.

## Figures and Tables

**Table 1:** FDA-approved drugs available for the therapy of high-grade gliomas (HGG).

FDA-Approved Drug	Mechanism	Year of Approval	Reference
Lomustine	Non-selective alkylating agent induces the crosslinking of DNA/RNA in dividing cells, thereby initiating cell apoptosis	1976	[99]
Carmustine	Binds to and alters the activity of glutathione reductase.	1977	[100]
Carmustine wafer implants	Binds to and alters the activity of glutathione reductase.	1996 & 2003	[101]
Temozolomide (TMZ)	A non-specific alkylating agent activates mismatch repair in DNA through methylation at the O6 position of guanine.	2005	[102]
Bevacizumab (BVZ)	A targeted healing antibody that attaches to and hinders the activity of the VEGF protein within tumor cells.	2009	[103]
